# CGMD: An integrated database of cancer genes and markers

**DOI:** 10.1038/srep12035

**Published:** 2015-07-10

**Authors:** Jangampalli Adi Pradeepkiran, Sri Bhashyam Sainath, Konidala Kramthi Kumar, Lokanada Balasubramanyam, Kodali Vidya Prabhakar, Matcha Bhaskar

**Affiliations:** 1 Division of Animal Biotechnology, Department of Zoology, Sri Venkateswara University, Tirupati-517502, Andhra Pradesh, India; 2 Department of Biotechnology, Vikrama Simhapuri University, Nellore-524 003, Andhra Pradesh, India; 3 CIMAR/CIIMAR, Centro Interdisciplinar de Investigação Marinha e Ambiental, Universidade do Porto, Rua dos Bragas, 177, 4050-123 Porto, Portugal

## Abstract

Integrating cancer genes and markers with experimental evidence might provide valuable information for the further investigation of crosstalk between tumor genes and markers in cancer biology. To achieve this objective, we developed a database known as the Cancer Gene Marker Database (CGMD), which integrates data on tumor genes and markers based on experimental evidence. The major goal of CGMD is to provide the following: 1) current systematic treatment approaches and recent advances in different cancer treatments; 2) the aggregation of different genes and markers by their molecular characteristics and pathway associations; and 3) free access to the data compiled by CGMD at http://cgmd.in/. The database consists of 309 genes and 206 markers, as well as a list of 40 different human cancers, with detailed descriptions of all characterized markers. CGMD provides complete cancer annotations and molecular descriptions of cancer genes and markers such as CpG islands, promoters, exons, PDB structures, active sites and domains.

A hallmark of cancer is abnormal cell proliferation that eventually leads to an imbalance between viable and dead cells. It is well accepted that genetic changes are one of the important causes of cancer^1^; however, the exact root cause of these genetic changes is not well defined. It has been suggested that mutations or alterations in the chromosomal make up of cells leads to changes in genomic architecture that result in cancer^2^. Recent evidence also indicates that mutations in more than 1% of human genes contribute to human cancer^3^. Moreover, these mutations are responsible for the switching of proto-oncogenes to oncogenes and/or the loss of function of tumor suppressor genes. Proto-oncogenes are normal genes that code for proteins regulating cell proliferation, differentiation and apoptosis^4^; thus, even small changes due to point mutations and/or chromosomal aberrations such as translocations, duplications, or deletions can convert normal proto-oncogenes into oncogenes. It has also been claimed that mutations in regulatory regions, especially in promoter sequences, and/or epigenetic mechanisms^5^, are the primary causes of various cancers. Mutations in promoter regions alter gene regulatory mechanisms and epigenetic changes such as hypo-or hyper-methylation might leads to chromosomal instability, altered expression and transcriptional silencing of tumor suppressor genes that may turns to tumor development^6^. Recently, it has been shown that non-protein-coding RNAs can act as tumor suppressor genes by controlling cell proliferation and apoptosis at the post-transcriptional level during neoplasm development^7^.It appears that mutations in target genes that are involved in the regulation of cell-proliferation lead to cancer. Therefore, understanding the basic causes of cancer at the cellular and molecular levels, i.e., the genes involved, their translated products (proteins), and their roles in biochemical mechanisms, might provide valuable insights.

Many research groups have used high-throughput strategies to show that the expression of tumor suppressor genes changes with respect to cancer stage and type. It is evident that, in addition to tumor genes, data on tumor markers contribute to a full picture of cancer stages and types. Thus, tumor markers will provide invaluable clues for identifying genes that are highly expressed in tumors but not in normal adult tissue. However, freely available databases including both tumor genes and markers have not yet been developed. Therefore, a prime concern is in integrating the available experimental data related to tumor genes at the genomic, transcriptomic and proteomic levels, which is important to fully understand the genetic alterations in cancer. To accomplish this task, a systematic integration of experimental evidence is required that carefully catalogs the known cancer genes and markers from a diverse accumulation of literature and that evaluates their consistency. Currently, there are two databases available, a database system for tumor suppressor genes (TSGDB)^8^ and a web resource for tumor suppressor genes (TSgene)^9^. TSGDB is based on only experimental evidence, whereas TSgene is a comprehensive database with data taken from UniProtKB^10^, the Tumor Associated Gene (TAG) database and PubMed abstracts. Here, we have developed a database known as the Cancer Gene Marker Database (CGMD) that includes information related to both tumor suppressor genes and tumor markers by integrating experimental evidence (http://www.ncbi.nlm.nih.gov/pubmed/) with cancer-related web-based tools. The CGMD consists of literature-supported data from well-established databases such as Kyoto Encyclopedia of Genes and Genomes (KEGG) genes^11^, National Center for Biotechnology Information (NCBI) catalogs^12^, and UniProtKB^10^. It covers a broad range of literature-based information, advances in cancer treatments, and updated information on characterized cancer genes and markers at the genomic and proteomic levels based on large-scale experimental evidence. Furthermore, we manually collected and curated experimental data from high-throughput screening in a systematic way in CGMD. The current version of CGMD includes 309 cancer genes and 206 markers, 515 genes and markers in total, corresponding to 40 different cancers with genetic and marker data. Moreover, CGMD encompasses different informational databases related to cancer genes. For example, the KEGG entry, position of the gene, CpG islands, promoter regions, and exons of different genes and markers involved in each cancer at the level of molecular aspects are included. For accuracy and quick response for the user, we developed a MySQL database which will help users access the data. The fundamental objective of CGMD is to collect and integrate cancer genes and markers from different databases and to provide elaborate and detailed annotation information for these cancer genes and markers. This database simply provides cross-linking of multiple information resource in a searchable user interface. This freely available database is user friendly and, at the same time, acts as a platform for a better understanding of the molecular framework of cancer genes and markers by providing reliable information that will be helpful to the cancer research community and for medical and diagnostic developments.

## Results

### Key entities in CGMD

**Figure 1:**
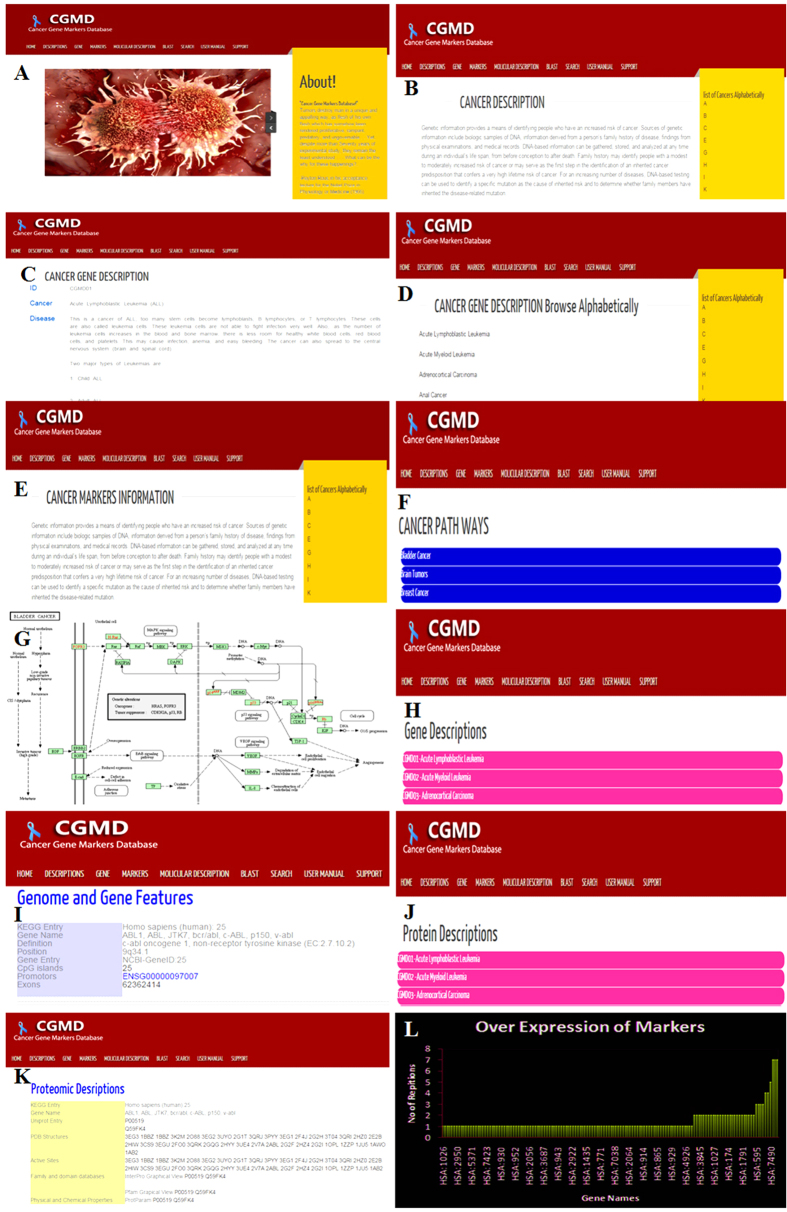
Web interface of the CGMD. (**A**) Basic home page displaying the information in CGMD. (**B**) Different cancer information displayed as a list from A to Z. (**C**) A typical literature highlight with supporting keywords for browsing alphabetically. (**D**) Gene description profile. (**E**) Query interface. (**F**) Browser for various cancer pathway types. (**G**) KEGG pathway mapped with CGMD (color-marked). (**H**) Gene description list for browsing. (**I**) provided genome and gene features. (**J**) Description list types for browsing (protein-coding). (**K**) Provided proteomic description features. (**L**) Over-expressed markers list in CGMD database.

The comprehensive CGMD database homepage is shown in (Fig. 1A). The homepage includes the tabs ‘Description,’ ‘Genes,’ ‘Markers,’ and ‘Molecular descriptions.’ Each tab is labeled separately to represent the stored information related to that tab. Clicking the tab ‘Description’ displays a list of cancers alphabetically from A to Z; by selecting a particular letter, the user can see a list of cancers beginning with that letter (Fig. 1B,C). This list was prepared from updated information from the National Cancer Institute (NCI)^13^. This tab includes the general information about each cancer, such as disease information, symptoms, pathogenicity, advances in treatments, and up-to-date literature evidence. The second tab of the database, the ‘Genes’ tab, is supported by the literature on nearly 309 genes associated with 40 types of cancers and their respective sequences (Fig. 1D,E). In the next tab, ‘Markers,’ almost 206 markers for various cancers, and their protein sequences are sequentially listed. Finally, we included the important aspects of CGMD in the ‘Molecular descriptions’ tab. This tab includes three contents: the ‘pathways,’ ‘gene description,’ and ‘protein description’ of each gene or marker (Fig. 1F,G). By clicking ‘pathways,’ a list of cancer markers and their pathways are displayed along with the number of tumor suppressor genes involved and their detailed descriptions with respect to different types of cancers (Fig. 1H). Next to the pathways tab, the user finds a gene description tab which displays a new navigation window that includes all associated cancers and their markers, providing detailed information for each cancer gene (Fig. 1I). Clicking on the Gene ID number displays the preloaded, annotated, analyzed data and cross-linked detailed information about the gene (Fig. 1I). To obtain the marker’s information the user simply clicks the Marker ID number. By clicking ‘protein description,’ a new navigation window is displayed with preloaded protein information that appears for each respective marker by clicking the Marker ID number (Fig. 1J,K). Finally, we have made a simple bar chart for over-expressed markers and genes (Fig. 1L).

### Inference about CGMD

The CGMD resource tool is user friendly. The resource tool includes tumor genes and tumor markers with a particular identity (ID) code that is intended to clearly distinguish genes and markers. Moreover, the CGMD-IDs have been cross-linked with Entrez ID codes, allowing them to be located in the genome based on the original literature source. Linking a variety of web resources for cancer genes and markers and extracting the output from various online tools from the database is manageable for the user.

### Implementation and future perspectives

CGMD provides information related to cancer genes and markers by integrating several types of data including genomic, proteomic, and active site interaction data. The rationale for the development of CGMD is to provide a platform to better understand the tumor genes at the molecular level in a way that is useful for therapeutic applications. Finally, we will continue on collection and curation of tumor genes and markers information and update the database by integrating the new experimental evidences related to tumor genes or markers accordingly. This will finally increase the specificity of pathways related to the tumor genes and markers for better understanding prognosis, prevention and for the treatment of cancer.

## Discussion

The major goal of the present database is to collect, integrate and maintain a high quality cancer gene marker database (CGMD), which serves as a comprehensive, fully categorized and accurately annotated gene markers of cancer. The database provides a good platform for cross-references and querying interfaces. The database is freely available to the public and sustains the basic and advanced cancer research for better perceptive molecular features of cancer genes and markers to prevent cancer. Cancer Gene Marker Database (CGMD) is an interface that provides valuable information about cancer genes linked to their experimental evidences and also acts as a platform to search cancer genes which acts as markers with specific mutations or with altered binding sites within the genes. CGMD is a user friendly tool which includes various types of mutations. The characterized mutation is considered separately as a mutation of a marker and as the corresponding gene sequence. For example breast cancer susceptibility mutation BRCA contains two genes; in CGMD, BRCA is included as cancer mutation and each of characterized genes BRCA1 and BRCA2 are considered as two independent genes. First, we align the cancer genes by searching respective keywords in the NCBI from a pool of sequences; we then isolate the respective sequence IDs in KEGG disease and create a short list that gives accurate data of the cancer genes. Each Entrez ID is listed separately and cross-checked against PubMed abstracts. Care has been taken not to overlap the cancer gene sequences with the marker sequences. Individual unique sets of both cancers genes and genes with markers (specific mutations or binding sites within genes) are prepared and used in the next step of the analysis. Each cancer sequence ID is then used as the key element to search and retrieve information on both cancer genes and markers from various genomic and proteomic tools. The analyzed output data are again cross-checked with the raw sequence data of cancer genes and markers to minimize error. After checking all of these steps, we manually draft separate genomic and proteomic data sets for both the cancer genes and the markers. Finally, we pool the information of nearly 2265 promoters, exons, and potential frames of translation products for each cancer gene. In the proteomic approach, we list all 2634 possible Protein Data Bank (PDB) structures from KEGG disease, domain descriptions, and the corresponding active sites, etc. For a better description of the functional annotations of the cancer genes and markers in CGMD, we provide the basic information as well as extensive molecular features of the cancer genes and markers (Table 1). Basic information on cancer genes obtained from UniProt entries and cross-linking databases, such as PDBsum, provides the core features of the cancer proteins. We also cross linked the data extensively with the KEGG Pathway database to include the cancer markers’ functional pathways. Finally, the protein super family characterizations of the cancer markers were obtained from the Pfam database^14^.

**Figure 2:**
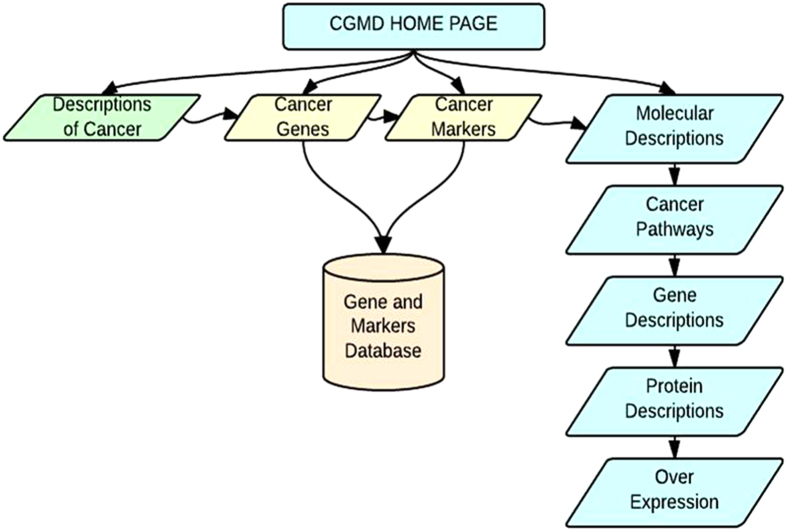
Schematic illustration of detailed integration of components associated with CGMD.

Overall, among the cancer markers and genes in the database, approximately 515 CpG islands were detected with conserved regions of sequence that can be protected against incidental errors in the genome^15^. We included the promoters, the distinct regulatory sites that are the functional switches of genes and that may be turned on or off by tuning the structural features of each chromosome^16^. Exon regions provide evidence of how exactly carcinogenic sequences may encode pathological protein products, as they help to predict cancer protein sequences in all possible reading frames^17^. The BLAST feature provides quick searches of corresponding cancer genes and markers in sequence databases. The database provides the PDB structures and PDB active sites from the PDB and PDBsum databases^18^. CGMD analyzes the physicochemical properties, such as molecular weight, pI, amino acid composition, instability index, protein stability, and aliphatic and grand average of hydropathicity (GRAVY), of proteins encoded by cancer genes and markers using the ProtParam tool^19^. The detailed contents of CGMD are illustrated in (Fig. 2).

## Methods

### Design and Implementation

**Figure 3:**
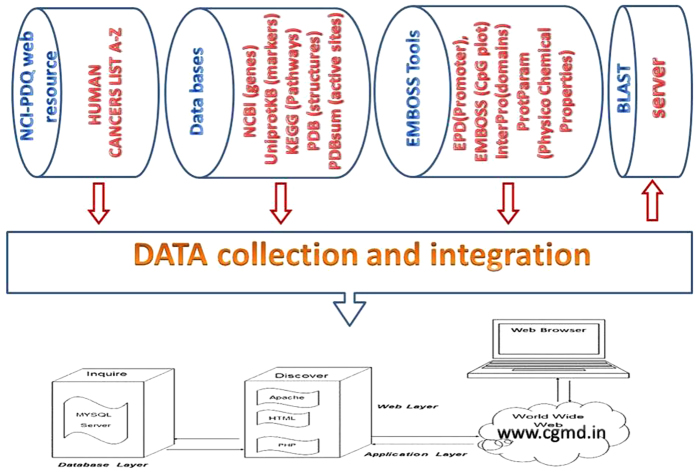
Flow-chart depicts the major aspects of CGMD database such as integration of genomic and proteomic components of different cancers.

We designed a MySQL database to store all data and a front end PHP tool to interface with the user for data retrieval and browsing. The database infrastructure enabled efficient storage and retrieval, and this database allows different resources to communicate with each other and also a good platform to access the information through PhpMyAdmin and JavaScript. An overview of work flow chart and the database architecture is shown in (Fig. 3). We first collected the cancer genes and markers from public databases such as NCBI, UniProtKB and KEGG disease and organized them systematically. From NCBI, UniProtKB and KEGG disease and we retrieved 309 genes (humans) and 206 markers (humans), using the key words cancer genes and cancer markers, respectively. However, none of the data sources provided original literature support to retrieve specific cancer genes and corresponding markers. Furthermore, literature-based information about cancer genes and markers forms concrete support to understand the mechanisms underlying the development of cancer which in turn at least in part seems crucial for better prognosis and treatment of cancer. Therefore, it is apparent that integration of the cancer genes and markers along with literature support is of prime concern. Thus, we developed a database which comprehends the literature-based information of cancer genes and markers.

Initially, we listed different types of cancers from the NCI-PDQ web resource^13^, then queried each cancer marker sequence in the KEGG database, and eventually characterized the sequences. All the sequences were cross-checked for PubMed literature evidence. Subsequently, a manual analysis process was performed to analyze the data pool at the molecular level using online bioinformatics tools. CGMD also contains a tab which links BLASTP to help users for protein sequence alignments [BLAST tab: www.Blastp program (v2.2.26)] perform sequence alignments. Moreover, for CGMD to provide reliable and detailed extensive information related to cancer markers, the Entrez and PubMed biomedical literature search systems and online sequence analysis tools were used as a source to analyze the data and sequentially organized the advanced information related to cancer genes and markers.

## Additional Information

**How to cite this article**: Jangampalli Adi, P. *et al.* CGMD: An integrated database of cancer genes and markers. *Sci. Rep.* **5**, 12035; doi: 10.1038/srep12035 (2015).

## Figures and Tables

**Table 1:** Components of CGMD database integrated with cancer genes and markers retrieved from different public resources.

S.No	Database Features	CGMD integration In number	Resource
1	Cancer genes	309	NCBI
2	Cancer markers	206	NCBI
3	Uniprot sequences	515	Uniprot
4	CpG islands	515	EMBOSS
5	Promoters	515	Interpro
6	Exons	515	EMBOSS
7	PDB Structures	2634	RCSB(PDB)
8	PDB Active sites	2634	PDBsum
9	Pfam domines	206	Pfam
10	Physico chemical properties	206	Prot param
